# Supplementary material for: A Critical View of the Application of the APEX Software (Aqueous Photochemistry of Environmentally-Occurring Xenobiotics) to Predict Photoreaction Kinetics in Surface Freshwaters
Source: Molecules. 2019 Dec 18;25(1):9. doi: 10.3390/molecules25010009 (PMC7017383; doi:10.3390/molecules25010009)
Supplement: Supplementary file 1 [file molecules-25-00009-s001.zip › 3.2.4_gcc-4.4.0.zip!/3.2.4_gcc-4.4.0/3.2.4_gcc-4.4.0/doc/octave/html/interpreter/Matrix-Factorizations.html]

Matrix Factorizations - Untitled


Next: Functions of a Matrix,
Previous: Basic Matrix Functions,
Up: Linear Algebra


---

### 18.3 Matrix Factorizations

— Loadable Function: r = **chol** (a)  
— Loadable Function: [r, p] = **chol** (a)  
— Loadable Function: [r, p, q] = **chol** (s)  
— Loadable Function: [r, p, q] = **chol** (s, 'vector')  
— Loadable Function: [l, ...] = **chol** (..., 'lower')  
> Compute the Cholesky factor, r, of the symmetric positive definite
> matrix a, where
>
> ```
>           r' * r = a.
> ```
>
> Called with one output argument `chol` fails if a or s is
> not positive definite. With two or more output arguments p flags
> whether the matrix was positive definite and `chol` does not fail. A
> zero value indicated that the matrix was positive definite and the r
> gives the factorization, and p will have a positive value otherwise.
>
> If called with 3 outputs then a sparsity preserving row/column permutation
> is applied to a prior to the factorization. That is r
> is the factorization of a`(`q`,`q`)` such that
>
> ```
>           r' * r = q' * a * q.
> ```
>
> The sparsity preserving permutation is generally returned as a matrix.
> However, given the flag 'vector', q will be returned as a vector
> such that
>
> ```
>           r' * r = a (q, q).
> ```
>
> Called with either a sparse or full matrix and using the 'lower' flag,
> `chol` returns the lower triangular factorization such that
>
> ```
>           l * l' = a.
> ```
>
> In general the lower triangular factorization is significantly faster for
> sparse matrices.
>
>
>
> **See also:** cholinv, chol2inv.

— Loadable Function: **cholinv** (a)  
> Use the Cholesky factorization to compute the inverse of the
> symmetric positive definite matrix a.
>
>
>
> **See also:** chol, chol2inv.

— Loadable Function: **chol2inv** (u)  
> Invert a symmetric, positive definite square matrix from its Cholesky
> decomposition, u. Note that u should be an upper-triangular
> matrix with positive diagonal elements. `chol2inv (`u`)`
> provides `inv (`u`'*`u`)` but it is much faster than
> using `inv`.
>
>
>
> **See also:** chol, cholinv.

— Loadable Function: [R1, info] = **cholupdate** (R, u, op)  
> Update or downdate a Cholesky factorization. Given an upper triangular
> matrix R and a column vector u, attempt to determine another
> upper triangular matrix R1 such that
>
> - R1'\*R1 = R'\*R + u\*u'
>   if op is "+"- R1'\*R1 = R'\*R - u\*u'
>     if op is "-"
>
> If op is "-", info is set to
>
> - 0 if the downdate was successful,- 1 if R'\*R - u\*u' is not positive definite,- 2 if R is singular.
>
> If info is not present, an error message is printed in cases 1 and 2.
>
>
>
> **See also:** chol, qrupdate.

— Loadable Function: [R1, info] = **cholinsert** (R, j, u)  
> Given a Cholesky factorization of a real symmetric or complex hermitian
> positive definite matrix A = R'\*R, R upper triangular,
> return the Cholesky factorization of
> A1, where A1(p,p) = A, A1(:,j) = A1(j,:)' = u and
> p = [1:j-1,j+1:n+1]. u(j) should be positive.
> On return, info is set to
>
> - 0 if the insertion was successful,- 1 if A1 is not positive definite,- 2 if R is singular.
>
> If info is not present, an error message is printed in cases 1 and 2.
>
>
>
> **See also:** chol, cholupdate, choldelete.

— Loadable Function: R1 = **choldelete** (R, j)  
> Given a Cholesky factorization of a real symmetric or complex hermitian
> positive definite matrix A = R'\*R, R upper triangular,
> return the Cholesky factorization of A(p,p), where p = [1:j-1,j+1:n+1].
>
>
>
> **See also:** chol, cholupdate, cholinsert.

— Loadable Function: R1 = **cholshift** (R, i, j)  
> Given a Cholesky factorization of a real symmetric or complex hermitian
> positive definite matrix A = R'\*R, R upper triangular,
> return the Cholesky factorization of
> A(p,p), where p is the permutation   
> `p = [1:i-1, shift(i:j, 1), j+1:n]` if i < j   
> or   
> `p = [1:j-1, shift(j:i,-1), i+1:n]` if j < i.   
>
>
>
>
> **See also:** chol, cholinsert, choldelete.

— Loadable Function: h = **hess** (a)  
— Loadable Function: [p, h] = **hess** (a)  
> Compute the Hessenberg decomposition of the matrix a.
>
> The Hessenberg decomposition is usually used as the first step in an
> eigenvalue computation, but has other applications as well (see Golub,
> Nash, and Van Loan, IEEE Transactions on Automatic Control, 1979). The
> Hessenberg decomposition is
> `p * h * p' = a` where `p` is a square unitary matrix
> (`p' * p = I`, using complex-conjugate transposition) and `h`
> is upper Hessenberg (`i >= j+1 => h (i, j) = 0`).

— Loadable Function: [l, u, p] = **lu** (a)  
— Loadable Function: [l, u, p, q] = **lu** (s)  
— Loadable Function: [l, u, p, q, r] = **lu** (s)  
— Loadable Function: [...] = **lu** (s, thres)  
— Loadable Function: y = **lu** (...)  
— Loadable Function: [...] = **lu** (..., 'vector')  
> Compute the LU decomposition of a. If a is full subroutines from
> lapack are used and if a is sparse then UMFPACK is used. The
> result is returned in a permuted form, according to the optional return
> value p. For example, given the matrix `a = [1, 2; 3, 4]`,
>
> ```
>           [l, u, p] = lu (a)
> ```
>
> returns
>
> ```
>           l =
>           
>             1.00000  0.00000
>             0.33333  1.00000
>           
>           u =
>           
>             3.00000  4.00000
>             0.00000  0.66667
>           
>           p =
>           
>             0  1
>             1  0
> ```
>
> The matrix is not required to be square.
>
> Called with two or three output arguments and a spare input matrix,
> then lu does not attempt to perform sparsity preserving column
> permutations. Called with a fourth output argument, the sparsity
> preserving column transformation Q is returned, such that
> p `*` a `*` q `=` l `*` u.
>
> Called with a fifth output argument and a sparse input matrix, then
> lu attempts to use a scaling factor r on the input matrix
> such that p `* (`r `\` a`) *` q `=` l `*` u.
> This typically leads to a sparser and more stable factorization.
>
> An additional input argument thres, that defines the pivoting
> threshold can be given. thres can be a scalar, in which case
> it defines UMFPACK pivoting tolerance for both symmetric and unsymmetric
> cases. If thres is a two element vector, then the first element
> defines the pivoting tolerance for the unsymmetric UMFPACK pivoting
> strategy and the second the symmetric strategy. By default, the values
> defined by `spparms` are used and are by default `[0.1, 0.001]`.
>
> Given the string argument 'vector', lu returns the values of p
> q as vector values, such that for full matrix, a `(`p`,:) =` l `*` u, and r`(`p`,:) *` a `(:,` q`) =` l `*` u.
>
> With two output arguments, returns the permuted forms of the upper and
> lower triangular matrices, such that a `=` l `*` u.
> With one output argument y, then the matrix returned by the lapack
> routines is returned. If the input matrix is sparse then the matrix l
> is embedded into u to give a return value similar to the full case.
> For both full and sparse matrices, lu looses the permutation
> information.

— Loadable Function: [q, r, p] = **qr** (a)  
— Loadable Function: [q, r, p] = **qr** (a, '0')  
> Compute the QR factorization of a, using standard lapack
> subroutines. For example, given the matrix `a = [1, 2; 3, 4]`,
>
> ```
>           [q, r] = qr (a)
> ```
>
> returns
>
> ```
>           q =
>           
>             -0.31623  -0.94868
>             -0.94868   0.31623
>           
>           r =
>           
>             -3.16228  -4.42719
>              0.00000  -0.63246
> ```
>
> The `qr` factorization has applications in the solution of least
> squares problems
>
> ```
>           min norm(A x - b)
> ```
>
> for overdetermined systems of equations (i.e.,
> `a`
> is a tall, thin matrix). The QR factorization is
> `q * r = a` where `q` is an orthogonal matrix and `r` is
> upper triangular.
>
> If given a second argument of '0', `qr` returns an economy-sized
> QR factorization, omitting zero rows of R and the corresponding
> columns of Q.
>
> If the matrix a is full, the permuted QR factorization
> `[`q`,` r`,` p`] = qr (`a`)` forms the QR factorization
> such that the diagonal entries of `r` are decreasing in magnitude
> order. For example,given the matrix `a = [1, 2; 3, 4]`,
>
> ```
>           [q, r, p] = qr(a)
> ```
>
> returns
>
> ```
>           q =
>           
>             -0.44721  -0.89443
>             -0.89443   0.44721
>           
>           r =
>           
>             -4.47214  -3.13050
>              0.00000   0.44721
>           
>           p =
>           
>              0  1
>              1  0
> ```
>
> The permuted `qr` factorization `[q, r, p] = qr (a)`
> factorization allows the construction of an orthogonal basis of
> `span (a)`.
>
> If the matrix a is sparse, then compute the sparse QR factorization
> of a, using CSparse. As the matrix Q is in general a full
> matrix, this function returns the Q-less factorization r of
> a, such that r `= chol (`a`' *` a`)`.
>
> If the final argument is the scalar `0` and the number of rows is
> larger than the number of columns, then an economy factorization is
> returned. That is r will have only `size (`a`,1)` rows.
>
> If an additional matrix b is supplied, then `qr` returns
> c, where c `=` q`' *` b. This allows the
> least squares approximation of a `\` b to be calculated
> as
>
> ```
>           [c,r] = spqr (a,b)
>           x = r \ c
> ```

— Loadable Function: [Q1, R1] = **qrupdate** (Q, R, u, v)  
> Given a QR factorization of a real or complex matrix
> A = Q\*R, Q unitary and
> R upper trapezoidal, return the QR factorization
> of A + u\*v', where u and v are
> column vectors (rank-1 update) or matrices with equal number of columns
> (rank-k update). Notice that the latter case is done as a sequence of rank-1 updates;
> thus, for k large enough, it will be both faster and more accurate to recompute
> the factorization from scratch.
>
> The QR factorization supplied may be either full
> (Q is square) or economized (R is square).
>
>
>
> **See also:** qr, qrinsert, qrdelete.

— Loadable Function: [Q1, R1] = **qrinsert** (Q, R, j, x, orient)  
> Given a QR factorization of a real or complex matrix
> A = Q\*R, Q unitary and
> R upper trapezoidal, return the QR factorization of
> [A(:,1:j-1) x A(:,j:n)], where u is a column vector to be
> inserted into A (if orient is `"col"`), or the
> QR factorization of [A(1:j-1,:);x;A(:,j:n)], where x
> is a row vector to be inserted into A (if orient is
> `"row"`).
>
> The default value of orient is `"col"`.
> If orient is `"col"`,
> u may be a matrix and j an index vector
> resulting in the QR factorization of a matrix B such that
> B(:,j) gives u and B(:,j) = [] gives A.
> Notice that the latter case is done as a sequence of k insertions;
> thus, for k large enough, it will be both faster and more accurate to recompute
> the factorization from scratch.
>
> If orient is `"col"`,
> the QR factorization supplied may be either full
> (Q is square) or economized (R is square).
>
> If orient is `"row"`, full factorization is needed.
>
>
>
> **See also:** qr, qrupdate, qrdelete.

— Loadable Function: [Q1, R1] = **qrdelete** (Q, R, j, orient)  
> Given a QR factorization of a real or complex matrix
> A = Q\*R, Q unitary and
> R upper trapezoidal, return the QR factorization of
> [A(:,1:j-1) A(:,j+1:n)], i.e., A with one column deleted
> (if orient is "col"), or the QR factorization of
> [A(1:j-1,:);A(:,j+1:n)], i.e., A with one row deleted (if
> orient is "row").
>
> The default value of orient is "col".
>
> If orient is `"col"`,
> j may be an index vector
> resulting in the QR factorization of a matrix B such that
> A(:,j) = [] gives B.
> Notice that the latter case is done as a sequence of k deletions;
> thus, for k large enough, it will be both faster and more accurate to recompute
> the factorization from scratch.
>
> If orient is `"col"`,
> the QR factorization supplied may be either full
> (Q is square) or economized (R is square).
>
> If orient is `"row"`, full factorization is needed.
>
>
>
> **See also:** qr, qrinsert, qrupdate.

— Loadable Function: [Q1, R1] = **qrshift** (Q, R, i, j)  
> Given a QR factorization of a real or complex matrix
> A = Q\*R, Q unitary and
> R upper trapezoidal, return the QR factorization
> of A(:,p), where p is the permutation   
> `p = [1:i-1, shift(i:j, 1), j+1:n]` if i < j   
> or   
> `p = [1:j-1, shift(j:i,-1), i+1:n]` if j < i.   
>
>
>
>
> **See also:** qr, qrinsert, qrdelete.

— Loadable Function: lambda = **qz** (a, b)  
> Generalized eigenvalue problem A x = s B x,
> QZ decomposition. There are three ways to call this function:
>
> 1. `lambda = qz(A,B)`
>
>    Computes the generalized eigenvalues
>    lambda
>    of (A - s B).- `[AA, BB, Q, Z, V, W, lambda] = qz (A, B)`
>
>      Computes qz decomposition, generalized eigenvectors, and
>      generalized eigenvalues of (A - sB)
>
>      ```
>                         A*V = B*V*diag(lambda)
>                         W'*A = diag(lambda)*W'*B
>                         AA = Q'*A*Z, BB = Q'*B*Z
>      ```
>
>      with Q and Z orthogonal (unitary)= I- `[AA,BB,Z{, lambda}] = qz(A,B,opt)`
>
>        As in form [2], but allows ordering of generalized eigenpairs
>        for (e.g.) solution of discrete time algebraic Riccati equations.
>        Form 3 is not available for complex matrices, and does not compute
>        the generalized eigenvectors V, W, nor the orthogonal matrix Q.
>
>        opt: for ordering eigenvalues of the GEP pencil. The leading block of the revised pencil contains all eigenvalues that satisfy: `"N"`: = unordered (default) `"S"`: = small: leading block has all |lambda| <=1 `"B"`: = big: leading block has all |lambda| >= 1 `"-"`: = negative real part: leading block has all eigenvalues in the open left half-plane `"+"`: = non-negative real part: leading block has all eigenvalues in the closed right half-plane
>
> Note: qz performs permutation balancing, but not scaling (see balance).
> Order of output arguments was selected for compatibility with matlab
>
>
>
>
> **See also:** balance, eig, schur.

— Function File: [aa, bb, q, z] = **qzhess** (a, b)  
> Compute the Hessenberg-triangular decomposition of the matrix pencil
> `(`a`,` b`)`, returning
> aa `=` q `*` a `*` z,
> bb `=` q `*` b `*` z, with q and z
> orthogonal. For example,
>
> ```
>           [aa, bb, q, z] = qzhess ([1, 2; 3, 4], [5, 6; 7, 8])
>                => aa = [ -3.02244, -4.41741;  0.92998,  0.69749 ]
>                => bb = [ -8.60233, -9.99730;  0.00000, -0.23250 ]
>                =>  q = [ -0.58124, -0.81373; -0.81373,  0.58124 ]
>                =>  z = [ 1, 0; 0, 1 ]
> ```
>
> The Hessenberg-triangular decomposition is the first step in
> Moler and Stewart's QZ decomposition algorithm.
>
> Algorithm taken from Golub and Van Loan, Matrix Computations, 2nd
> edition.

— Loadable Function: s = **schur** (a)  
— Loadable Function: [u, s] = **schur** (a, opt)  
> The Schur decomposition is used to compute eigenvalues of a
> square matrix, and has applications in the solution of algebraic
> Riccati equations in control (see `are` and `dare`).
> `schur` always returns
> `s = u' * a * u`
> where
> `u`
> is a unitary matrix
> (`u'* u` is identity)
> and
> `s`
> is upper triangular. The eigenvalues of
> `a` (and `s`)
> are the diagonal elements of
> `s`.
> If the matrix
> `a`
> is real, then the real Schur decomposition is computed, in which the
> matrix
> `u`
> is orthogonal and
> `s`
> is block upper triangular
> with blocks of size at most
> `2 x 2`
> along the diagonal. The diagonal elements of
> `s`
> (or the eigenvalues of the
> `2 x 2`
> blocks, when
> appropriate) are the eigenvalues of
> `a`
> and
> `s`.
>
> The eigenvalues are optionally ordered along the diagonal according to
> the value of `opt`. `opt = "a"` indicates that all
> eigenvalues with negative real parts should be moved to the leading
> block of
> `s`
> (used in `are`), `opt = "d"` indicates that all eigenvalues
> with magnitude less than one should be moved to the leading block of
> `s`
> (used in `dare`), and `opt = "u"`, the default, indicates that
> no ordering of eigenvalues should occur. The leading
> `k`
> columns of
> `u`
> always span the
> `a`-invariant
> subspace corresponding to the
> `k`
> leading eigenvalues of
> `s`.

— Function File: angle = **subspace** (a, B)  
> Determine the largest principal angle between two subspaces
> spanned by columns of matrices a and b.

— Loadable Function: s = **svd** (a)  
— Loadable Function: [u, s, v] = **svd** (a)  
> Compute the singular value decomposition of a
>
> ```
>           A = U*S*V'
> ```
>
> The function `svd` normally returns the vector of singular values.
> If asked for three return values, it computes
> U, S, and V.
> For example,
>
> ```
>           svd (hilb (3))
> ```
>
> returns
>
> ```
>           ans =
>           
>             1.4083189
>             0.1223271
>             0.0026873
> ```
>
> and
>
> ```
>           [u, s, v] = svd (hilb (3))
> ```
>
> returns
>
> ```
>           u =
>           
>             -0.82704   0.54745   0.12766
>             -0.45986  -0.52829  -0.71375
>             -0.32330  -0.64901   0.68867
>           
>           s =
>           
>             1.40832  0.00000  0.00000
>             0.00000  0.12233  0.00000
>             0.00000  0.00000  0.00269
>           
>           v =
>           
>             -0.82704   0.54745   0.12766
>             -0.45986  -0.52829  -0.71375
>             -0.32330  -0.64901   0.68867
> ```
>
> If given a second argument, `svd` returns an economy-sized
> decomposition, eliminating the unnecessary rows or columns of u or
> v.


— Function File: [housv, beta, zer] = **housh** (x, j, z)  
> Compute Householder reflection vector housv to reflect x
> to be the j-th column of identity, i.e.,
>
> ```
>           (I - beta*housv*housv')x =  norm(x)*e(j) if x(1) < 0,
>           (I - beta*housv*housv')x = -norm(x)*e(j) if x(1) >= 0
> ```
>
> Inputs
>
> x: vector j: index into vector z: threshold for zero (usually should be the number 0)
>
> Outputs (see Golub and Van Loan):
>
> beta: If beta = 0, then no reflection need be applied (zer set to 0) housv: householder vector

— Function File: [u, h, nu] = **krylov** (a, v, k, eps1, pflg)  
> Construct an orthogonal basis u of block Krylov subspace
>
> ```
>           [v a*v a^2*v ... a^(k+1)*v]
> ```
>
> Using Householder reflections to guard against loss of orthogonality.
>
> If v is a vector, then h contains the Hessenberg matrix
> such that `a*u == u*h+rk*ek'`, in which `rk =
> a*u(:,k)-u*h(:,k)`, and `ek'` is the vector
> `[0, 0, ..., 1]` of length `k`. Otherwise, h is
> meaningless.
>
> If v is a vector and k is greater than
> `length(A)-1`, then h contains the Hessenberg matrix such
> that `a*u == u*h`.
>
> The value of nu is the dimension of the span of the krylov
> subspace (based on eps1).
>
> If b is a vector and k is greater than m-1, then
> h contains the Hessenberg decomposition of a.
>
> The optional parameter eps1 is the threshold for zero. The
> default value is 1e-12.
>
> If the optional parameter pflg is nonzero, row pivoting is used
> to improve numerical behavior. The default value is 0.
>
> Reference: Hodel and Misra, "Partial Pivoting in the Computation of
> Krylov Subspaces", to be submitted to Linear Algebra and its
> Applications
